# Overcoming Oral Cavity Barriers for Peptide Delivery Using Advanced Pharmaceutical Techniques and Nano-Formulation Platforms

**DOI:** 10.3390/biomedicines13112735

**Published:** 2025-11-08

**Authors:** Ali A. Amer, Lewis Bingle, Amal Ali Elkordy, Cheng Shu Chaw

**Affiliations:** 1 School of Pharmacy and Pharmaceutical Sciences, Faculty of Health Sciences and Wellbeing, University of Sunderland, Sunderland SR1 3SD, UK; amal.elkordy@sunderland.ac.uk (A.A.E.); hs0cch@sunderland.ac.uk (C.S.C.); 2 School of Nursing and Health Sciences, Faculty of Health Sciences and Wellbeing, University of Sunderland, Sunderland SR1 3SD, UK; lewis.bingle@sunderland.ac.uk

**Keywords:** peptide delivery, buccal mucosa, nanoparticles, lipid-based carriers, mucosal permeability, surface modification, oral drug delivery, mucoadhesion, endocytic uptake, tight junction modulation, cell-penetrating peptides, nanocarrier system

## Abstract

Therapeutic peptides have gained significant attention due to their high specificity, potency, and safety profiles in treating various diseases. However, their clinical application via the oral route remains challenging. Peptides are inherently unstable in the gastrointestinal environment, where they are rapidly degraded by proteolytic enzymes and acidic pH, leading to poor bioavailability. Additionally, their large molecular size and hydrophilicity restrict passive diffusion across the epithelial barriers of the gastrointestinal tract. These limitations have traditionally necessitated parenteral administration, which reduces patient compliance and convenience. The oral cavity, comprising the buccal and sublingual mucosa, offers a promising alternative for peptide delivery. Its rich vascularization allows for rapid systemic absorption while bypassing hepatic first-pass metabolism. Furthermore, the mucosal surface provides a relatively permeable and accessible site for drug administration. However, the oral cavities also present significant barriers: the mucosal epithelium limits permeability, the presence of saliva causes rapid clearance, and enzymes in saliva contribute to peptide degradation. Therefore, innovative strategies are essential to enhance peptide stability, retention, and permeation in this environment. Nanoparticle-based delivery systems, including lipid-based carriers such as liposomes and niosomes, as well as polymeric nanoparticles like chitosan and PLGA, offer promising solutions. These nanocarriers protect peptides from enzymatic degradation, enhance mucoadhesion to prolong residence time, and facilitate controlled release. Their size and surface properties can be engineered to improve mucosal penetration, including through receptor-mediated endocytosis or by transiently opening tight junctions. Among these, niosomes have shown high encapsulation efficiency and sustained release potential, making them particularly suitable for oral peptide delivery. Despite advances, challenges remain in translating these technologies clinically, including ensuring biocompatibility, scalable manufacturing, and patient acceptance. Nevertheless, the oral cavity’s accessibility, combined with nanotechnological innovations, offers a compelling platform for personalized, non-invasive peptide therapies that could significantly improve treatment outcomes and patient quality of life.

## 1. Introduction

### Importance of Peptide-Based Therapeutics

There are certain categories of pharmacologically active ingredients, for example, hydrophobic drugs, enzymes, therapeutic proteins, hormones, antimicrobials, vaccines and peptides that face challenges when administered orally, which might affect their solubility and/or structural stability. Most of these active ingredients are extremely susceptible to hydrolysis, aggregation, or denaturation inside the human gastrointestinal tract (GIT) or on the shelf as commercially available pharmaceutical products. Colloidal systems including nano- or micro-particles could be engineered to maintain, protect, encapsulate and transport active macromolecules. For instance, some peptides could possibly be encapsulated and stabilized throughout storage and transition through the mouth and stomach, prior to release in the absorption sites of the small intestine. However, several significant technological obstacles must be addressed before these compounds can be efficiently administered orally [1].

Peptides are short polymers typically composed of fewer than 50 amino acids and generally have a molecular weight of less than 10 kDa. They represent a rapidly growing area in the development of new therapeutic agents. In addition, peptide exhibit unique pharmacokinetic properties and offers notable benefits as therapeutic agents Table 1 [2]. Proteins and peptides often demonstrate high biological activity per unit mass and can show good tissue penetration depending on their structure and delivery method [3]. Therapeutic peptides provide important pharmacological advantages. These include high target specificity and selectivity that can increase potency while reducing off-target toxicity, and a low propensity for drug–drug interactions because they are catabolized to amino acids rather than competing for small-molecule metabolic pathways [4]. These benefits are counterbalanced by practical and pharmacokinetic limitations. Manufacturing costs are typically higher than for small molecules because production is complex and requires specialized processes. Systemic exposure is often brief because peptides are rapidly cleared from the circulation and are susceptible to proteolytic degradation. [5]. Additionally, peptides have limited oral bioavailability due to their larger molecular weight and have different solubility in comparison to small drug molecules [6,7].

This review outlines strategies to deliver therapeutic peptides with a specific focus on the oral cavity. Barriers to oral uptake, including salivary wash-off, mucus interactions, tight junctions, and proteolysis are summarized, followed by advanced techniques that address them through mucoadhesion, mucus-penetration, permeation enhancement, and device integration. In addition to nano-formulation platforms (lipid vesicles, polymeric nanoparticles, niosomes, and hybrids) are assessed with attention to behavior in saliva, surface residence, and epithelial transport. key translational limits are noted, and practical design and reporting guidance is offered for buccal and sublingual delivery.

## 2. Modes of Peptide Administration

### 2.1. Parental Route

The most common routes for peptide delivery are the parenteral route, including intravenous and subcutaneous administration. This approach offers the major advantage of bypassing gastrointestinal enzymes and hepatic first-pass metabolism, thereby achieving higher systemic bioavailability of therapeutic peptides. However, subcutaneous and intramuscular delivery are still subject to a certain degree of pre-systemic degradation at the injection site. This occurs due to enzymatic activity within the interstitial space, where peptidases and proteases are present, as well as degradation processes within the lymphatic system [8].

Intravenous administration of therapeutic peptides delivers them directly into the systemic circulation, resulting in 100% bioavailability. In contrast, systemic bioavailability following subcutaneous or intramuscular administration can vary widely, typically ranging from 20% to 100%, depending on factors such as enzymatic degradation at the injection site, absorption kinetics, and lymphatic uptake [9]. The absorption kinetics of peptides following subcutaneous or intramuscular administration depend strongly on the physicochemical properties of the molecule. After injection, peptide drugs can be absorbed into the systemic circulation via both blood capillaries and lymphatic vessels. The relative contribution of these pathways is largely determined by molecular weight. Low-molecular-weight peptides (typically <1 kDa) are absorbed predominantly through blood capillaries, whereas larger molecules, particularly proteins exceeding 16–22 kDa, rely mainly on lymphatic uptake. As molecular weight increases, the lymphatic system becomes the primary route of absorption for peptide therapeutics. Considering that most therapeutic peptides fall within the molecular weight range of less than 10 kDa, it is generally expected that both the lymphatic system and blood vessels contribute to their absorption [10,11]. Usually, only a small portion of the dosage is absorbed by the lymphatic system via convective transport mechanisms.

Parenteral therapy for therapeutic peptide delivery is invasive and often unpleasant. Most peptides are delivered parenterally due to their susceptibility to enzymatic degradation and poor membrane permeability in the GIT. However, several examples, such as oral semaglutide and intranasal desmopressin demonstrate the growing potential of alternative delivery strategies through the oral route to improve patient compliance. Consequently, efforts were made to find alternative and non-invasive methods for administering these peptides, including oral, buccal (inside the cheek), pulmonary (via the lungs), intranasal (through the nose), and transdermal (through the skin) routes. The main limitations of peptides pharmacokinetics are their short half-life, primarily because they undergo proteolytic cleavage by enzymes such as peptidases and proteases, resulting in the breakdown of amino acids [2].

### 2.2. Oral Peptide Administration Directed to Intestinal Tract

**Figure 1:**
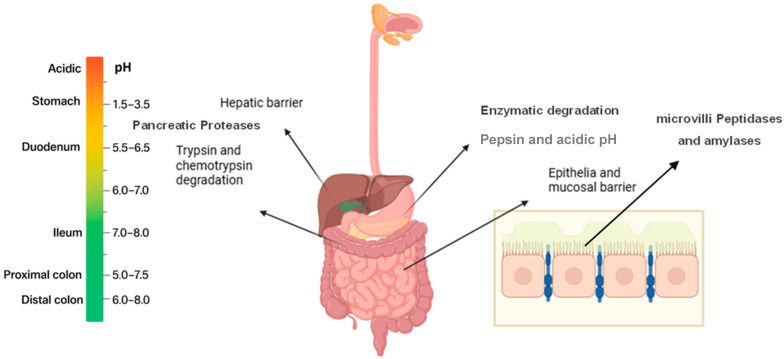
Physiological and chemical barriers to oral peptide drug delivery in the gastrointestinal tract. Created with BioRender.

Orally absorbed of therapeutic peptides meet several obstacles and barriers before reaching the systemic circulation. Physicochemical factors, such as molecule size, surface charge and density, as well as physiological barriers govern peptide absorption in the GIT [12]. Figure 1 represents factors and barriers encountered following oral administration of peptides.

The gastrointestinal pH extends across a wide range, starting from the acidic environment of the stomach (with pH 1.5 to 3.5 in fasting individuals and 3.0 to 5.0 after food ingestion) and extending to (pH 5.5 to 7) in the intestines [13,14]. Rapid fluctuations in pH within the gastrointestinal tract can significantly affect the stability of orally administered peptides. A notable change occurs as peptides move from the stomach to the duodenum, where the environment shifts from the highly acidic gastric environment to a near-neutral pH. Gastric juice, composed of hydrochloric acid, salts, and other components, contributes to this acidic environment. The surface charge of peptides is closely related to their isoelectric point, at which they carry no net charge. Variations in luminal pH modify the ionization states of amino acid residues present at the molecular surface, thereby affecting peptide solubility, aggregation behaviors, and interactions with the mucus layer. These changes in surface charge and density ultimately affect membrane permeability and absorption efficiency as peptides transit through the gut [12].

The pH-related chemical reactions, such as oxidation, hydrolysis, or deamidation, can subsequently cause a loss of peptide functionality. For example, the breakdown of proteins and peptides into their individual dipeptides, tripeptides, and amino acids occur in the acidic environment of the stomach. Meanwhile, deamidation, which converts glutamine and asparagine residues side chain amides into carboxylic acids, occurs at both low and near-neutral pH through different degradation mechanisms [14]. Additionally, amino acids like histidine, asparagine, tryptophan, cysteine, tyrosine, and methionine found in insulin are vulnerable to hydrolysis and oxidation when in solution. These reactions can especially take place at the asparagine residue and have been linked to the breaking of peptide bonds, ultimately leading to a decrease in biological activity [15,16,17].

Peptides also suffer from enzymatic degradation by proteases and peptidases that are secreted into the gut lumen, attached to the brush-border membrane, and within the cytosol [18,19]. For instance, pepsin is the first enzyme encountered with broad substrate selectivity in which its activity increases in the low pH of the stomach [20]. Pepsin’s activity decreases as it enters the duodenum, which has a higher pH value. The duodenum, jejunum, and ileum are three separate anatomical parts of the small intestine, that are generally identified as the principal location for the physiological absorption of foods. Particularly, the small intestine has significantly higher levels of enzymatic activity than any other parts of the GI tract [21,22].

The presence of pancreatic enzymes in the duodenum is particularly significant. These include endopeptidases such as trypsin and chymotrypsin, which cleave peptide bonds within the protein chain, and exopeptidases such as carboxypeptidase A and B, which cleave amino acid residues from the ends of the peptide chain [23]. Pancreatic juice is slightly alkaline, it boosts the pH of duodenum, activating other intestinal enzymes. The surface of intestinal epithelial cells is covered by the brush border, which consists of densely packed cellular extrusions called microvilli [24]. Enzymes including peptidases and amylases are present in the brush border membrane. Together, these enzymes also breaks down both proteins and peptides as well as starch and sucrose [25,26].

As the peptide drugs move along the GIT, they face cellular barriers during oral delivery. The small intestine is the main site for drug absorption. The epithelial lining of small intestine presents the next challenge if peptides survive in the biochemical assault and harsh environment in stomach, brush border and jejunum [27,28]. The epithelial lining is a complex structure containing several components, including the glycocalyx, mucus, intestinal microvilli, finger-like projections, and tight junctions, as well as an extensive sulphated mucopolysaccharide layer. These elements present a physiochemical barrier that strictly limits absorption of peptides [29,30]. Especially for hydrophilic molecules with a large molecular weight (>700 Da), diffusion across gut membranes, diffusion of these molecules across gut membrane is perturbed [31,32]. Finally, peptides that evade these obstacles are metabolized in the liver before reaching the systemic circulation. This has led to low and erratic oral bioavailability for orally administered peptides and current data suggest that only 1–2% of therapeutic peptide doses reach systemic circulation via conventional oral route [33]. Several attempts and approaches to overcome these challenges has been reviewed [19,34].

### 2.3. Peptide Administration via Oral Cavity

**Figure 2:**
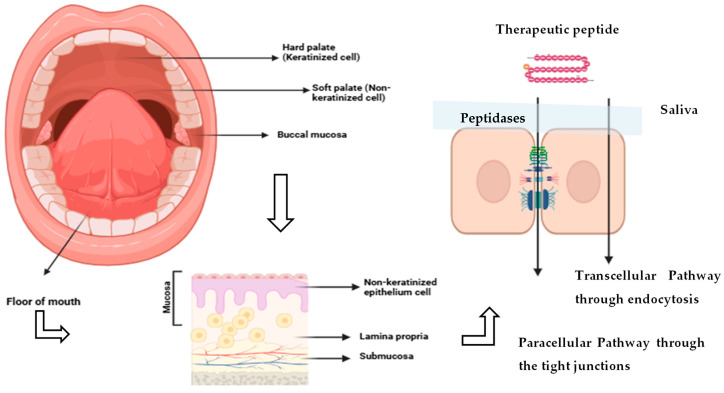
Oral cavity (**left**) and structure of oral mucosa (**middle**). Transport pathways for peptides (**right**). Created with BioRender.

Alternative non-invasive delivery routes such as the buccal and sublingual pathways have gained attention for systemic drug administration, particularly for macromolecules with poor oral bioavailability. The structure of the mouth cavity and oral mucosa is presented in Figure 2 (left and middle). The oral mucosa covers approximately 170 cm^2^, plays a critical role in drug delivery in the mouth, with its structure and properties at given sites influencing the absorption and effectiveness of medications [35]. The oral mucosa has two main layers: the lamina propria, which supplies blood vessels and support, and the outer stratified squamous epithelium, which protects the tissue (Figure 2 right). In areas such as the hard palate and gums, the epithelium is keratinized and arranged into four layers including basal, prickle, granular, and keratinized. This layered structure provides strength and durability but greatly reduces permeability, making these regions less suitable for drug absorption [36]. These areas account for about 25% of the mucosal surface. On the other hand, non-keratinized regions, particularly the buccal mucosa, are more flexible and provide a greater surface area for drug absorption.

The buccal mucosa is a key site for drug delivery, offering high permeability and dense vascularization that supports rapid molecular diffusion and absorption into the systemic circulation [37,38]. Oral drug absorption is strongly influenced by the structural characteristics of the epithelium, particularly its thickness and permeability. The buccal epithelium, lining the inner cheeks, provides a large absorptive surface with a thickness of approximately 500–600 μm. In contrast, the sublingual epithelium, located on the floor of the mouth, is much thinner (100–200 μm) but has a more restricted surface area. Despite this limitation, the sublingual region is characterized by superior permeability, rendering it an advantageous route for systemic drug administration [39,40,41].

Membrane coating granules (MCGs) in the superficial epithelial cells produce specific lipids that affect the mucosa’s permeability to drugs. In keratinized regions, MCGs produce non-polar lipids that enhance the barrier function, limiting drug penetration. In contrast, non-keratinized areas, such as buccal mucosa, contain MCGs that produce polar lipids, allowing for more selective permeability and greater potential for drug absorption. These structural and biochemical characteristics make the buccal mucosa an ideal site for localized or systemic peptide delivery, facilitating the absorption of drugs like analgesics, antihistamines, and hormone therapies directly into the bloodstream [42]. Two main routes of administration to the oral cavities are via the sublingual mucosa and through the buccal mucosa. These highly vascularized areas make up around 60% of the oral mucosa are ideal for systemic absorption. They are also widely used to treat localized oral disorders such as fungal infections, ulcers, and periodontal disease [43,44].

Saliva, a biological fluid found in the oral cavity, is secreted by submandibular, parotid and sublingual glands, in addition to tiny submucosal glands. This fluid, which is constantly created, dispersed, and eliminated, is essential to dental health. Among its prominent features are proteins with a variety of antibacterial qualities, the presence of electrolytes and organic compounds that control pH levels [45]. The amount of peptide molecules available at the absorption site is greatly impacted by the salivary renewal cycle. Due to the quick turnover of saliva, the peptides residence period in the oral cavity is shortened, which limits absorption. Furthermore, the pH and content of saliva affect the concentration and solubility of therapeutic peptides. The pH of oral fluid is slightly acidic but in disease states like gastroesophageal reflux disease, dental caries, the fluid becomes more acidic with a lowered pH [46,47]. Additionally, the flow rate of saliva, which varies throughout the day and alters with food consumption, affects the pH and composition of saliva. Increased salivary flow dilutes the medication, which may reduce its absorption and therapeutic impact [48].

Mucus is released by the salivary glands forming part of the oral fluids that deposit onto the epithelial cells. Water, enzymes, electrolytes, and mucins make up the majority of the mucus layer [49]. Mucins are glycoproteins with molecular weights typically ranging from 0.5 to 50 kDa. Together, these chemicals create a three-dimensional network that lubricates the oral mucosa, promotes cell migration between neighboring cells, and protects the mucosa underneath [50,51]. Mucins are heavily glycosylated, and their terminal sialic acids are ionized at physiological pH, giving the mucus network a net negative charge. This electrostatic environment can either hinder or facilitate peptide absorption, depending on peptide size, charge, hydrophobicity, and surface coating [52].

The tongue, a crucial organ in the oral cavity, makes up around 15% of the surface of the oral mucosa and is made up of skeletal muscle covered in a mucous membrane [53]. Movement of the tongue is facilitated by its extrinsic muscles [54]. During mastication, the tongue is crucial for manipulating food and swallowing, although it also serves as barrier to drug absorption.

Designing a drug delivery system for administration via the oral cavity requires a comprehensive knowledge of the anatomy and physiology of the mouth cavity as well as proficiency in formulation and manufacturing techniques to overcome obstacles in order to capitalize on the advantages of this route of administration (Table 2). Buccal dosage forms, for instance with flexible design platforms, are easy to apply and this convenience enhances patient acceptance and compliance. Buccal drug delivery also allows direct absorption of medications into the bloodstream through the highly vascularized oral mucosa, avoiding hepatic first-pass metabolism, and the harsh enzymatic and acidic conditions of the GIT, that can breakdown peptides [41].

## 3. Mechanisms of Peptide Uptake Across Oral Mucosa via Oral Cavity

Peptide absorption through the oral mucosa occurs via two principal pathways: transcellular (across epithelial cells) and paracellular (between cells) routes (Figure 2 right). However, the physicochemical characteristics of peptides, including large molecular weight, polar, and enzymatic lability substantially hinder their permeability and absorption [58,59,60]. The transcellular route favors lipophilic and low molecular weight molecules, as it requires partitioning into the lipid bilayer of epithelial membrane, hence most peptides exhibit poor affinity for this pathway [40,61]. While mechanisms such as endocytosis or transcytosis may permit limited peptide transport, these are low-capacity processes not sufficient for efficient delivery [62]. The paracellular route, though slightly more permissive in non-keratinized epithelium when compared to keratinized tissues, is also restrictive due to intercellular junctions that prevent movement of large hydrophilic molecules. In addition to passive diffusion, carrier-mediated transport systems in the oral mucosa may offer potential opportunities for peptide delivery. While nutrient-related transporters, such as glucose transporters (SGLT1, GLUT1–3) and amino acid transporters (LAT1, LAT2), are expressed in oral epithelial cells and could influence amino acid availability. Their contribution to peptide absorption across the oral mucosa remains largely unproven. Nevertheless, these transport systems represent potential targets for future strategies aimed at enhancing the delivery of peptide therapeutics via the oral mucosal route [61,62,63].

## 4. Challenges of Peptide Transport and Absorption via Oral Mucosa

Peptide absorption through the oral mucosa is severely restricted by their structural characteristics. Compounds with molecular weights above 500 Da generally show poor permeability, and most peptides, typically exceeding 1000 Da, fall well into this category. Their strong hydrophilicity limits membrane partitioning, while ionizable side chains often interact unfavorably with the mucosal surface, making passive diffusion largely ineffective. Moreover, enzymatic degradation by salivary and mucosal proteases such as aminopeptidases and esterases occurs before peptides can cross the epithelial barrier, further reducing their systemic bioavailability [64]. In addition to enzymatic and physicochemical barriers, the buccal mucosa also presents metabolic challenges. Although the cytochrome P450 enzyme system is present, its activity is relatively lower compared with the gastrointestinal tract. Importantly, the buccal mucosa exhibits reduced expression of P-glycoprotein (P-gp) efflux pumps compared with the intestinal mucosa. Since P-gp actively transports many substrates back into the lumen, this lower expression may confer a relative advantage for drug delivery via the buccal route. Consequently, despite the limitations associated with molecular size, hydrophilicity, and enzymatic degradation, the reduced efflux activity makes the buccal mucosa a comparatively favorable site for improving peptide absorption [65].

Moreover, peptides can be degraded in the oral cavity due to the variable pH of the saliva, unless supported by advanced formulation such as mucoadhesive systems, permeation enhancers, or nano-enabled delivery platforms like liposomes, niosomes and polymeric nanoparticles [66]. In addition, not all drugs are suitable for administration via the buccal route. Medications that cause local irritation or allergic reactions in the oral cavity, as well as those with an intensely bitter taste, unpleasant odor, or instability at salivary pH, are generally poor candidates for buccal delivery. Saliva also represents a critical variable influencing absorption. Its continuous secretion and renewal dilute the therapeutic peptide on the mucosal surface, significantly reducing the concentration available for uptake. Moreover, involuntary swallowing of saliva, the dosage form itself, or concomitant food and liquid intake can physically remove the drug from the absorption site, further lowering absorption efficiency. These factors may result in subtherapeutic plasma concentrations and reduced bioavailability [67]. studies with insulin demonstrate that only about 1–2% of a peptide dose delivered through conventional buccal or sublingual routes reaches systemic circulation, highlighting the considerable barriers that remain for effective peptide delivery via the oral mucosa [33,68]. Furthermore, discomfort and toxicity may result from extended interactions between some drug delivery methods and the buccal mucosa at the application site. In order to succeed, an innovative buccal peptide delivery system must effectively address these limitations.

## 5. Oral Mucosal Permeability of Peptides in the Oral Cavity

### 5.1. Peptide Molecular Weight and Buccal Mucosal Permeability

Molecular weight and size are the primary determinants of diffusivity through the buccal epithelium. Small molecules below 75–100 Da can readily permeate the mucosa, whereas permeability decreases sharply as molecular size increases, making most therapeutic peptides poorly absorbed via this route without incorporating any additives. A study performed by Hoogstraate et al. showed that neutral polysaccharides like fluorescein isothiocyanate-dextrans (FITC-dextrans) as model compounds for peptides and proteins exhibit reduced permeability as their molecular weight increases, particularly beyond 10 kDa [69]. Also, Fantini et al. (2022) showed that FITC-dextrans ≥ 70 kDa exhibited negligible passive permeation across porcine esophageal mucosa (buccal model), but co-administration with sodium taurocholate or caprylic acid significantly increased permeability, enabling transmucosal delivery of high-M.Wt dextrans [70]. Similarly, Keum et al. demonstrated that salmon calcitonin (M.Wt. 3.4 kDa), a model peptide drug, exhibited poor passive permeability, but when adding a cell-penetrating peptide (CPP) the buccal absorption increased by 5.5-fold in TR146 cell layers and an impressive ~93.7-fold in ex vivo porcine buccal mucosa, illustrating the deep effect of biochemical permeation enhancers [71].

In fact, buccal permeability of macromolecules is generally limited to peptides under 20–40 kDa, with sublingual mucosa sometimes permitting compounds up to 70 kDa with the use of penetration enhancers. Berka et al. investigated the passive permeability of different FITC-dextrans M.Wt grades and bovine serum albumin (BSA) across porcine sublingual mucosa. They found that the permeability of dextrans sharply decreased with increasing molecular weight, showing negligible transport above 10 kDa. However, BSA (~66 kDa) exhibited better permeability than similarly sized dextrans, likely due to differences in molecular structure and charge affecting interaction with the mucosal barrier [72]. Additionally, the study showed that applying the compounds using nanofiber mats significantly enhanced mucosal permeability by increasing local drug concentration and providing close contact with the mucosa, thus improving diffusion and absorption. highlights the potential of nanofiber-based delivery systems to overcome the sublingual mucosal barrier for peptides and proteins Table 3. Macromolecules like dextrans showed that the paracellular route is the major pathway through buccal epithelium [70]. Human data indicate that smaller peptides like protirelin (362 Da) and oxytocin (1007 Da) successfully permeate buccal tissue, whereas larger ones like buserelin (1239 Da) and calcitonin (3500 Da) do not, supporting the concept that molecules over 1000 Da and require permeability enhancers [73].

### 5.2. Conformation and Immunogenicity

Peptide drugs differ from conventional drugs as they have complex 3D structures that can change in solution depending on their size and pH media. In an in silico study, the membrane diffusion of conformationally flexible cyclic peptides was shown to depend on their dynamic interactions with lipid bilayer components. Initially, specific peptide residues interact with the polar headgroups and hydrophobic tails of the membrane lipids, allowing the peptide to insert partially into the bilayer. Once inserted, the peptide can adopt a “closed conformation,” in which polar and charged groups are shielded from the hydrophobic membrane core while hydrophobic residues interact favorably with the lipid tails. This structural rearrangement reduces energetic barriers, enabling the peptide to traverse the inner leaflet of the membrane and ultimately diffuse across the bilayer. The process highlights the importance of both peptide flexibility and conformational adaptability in overcoming the hydrophobic and steric challenges of lipid membranes, which is particularly relevant for designing peptides with improved mucosal permeability [74]. whereas, maintaining the native conformation during formulation and manufacturing processes is crucial because conformational changes can influence membrane permeability [31]. whereas modifying peptide structure or conjugating them with inert polymers like polyethylene glycol (PEG) or dextran not only helps reduce immunogenicity and protects them from enzymatic breakdown but also improves the mucosal permeability of peptides [75,76,77].

### 5.3. Physicochemical Peptide Properties (Solubility, Hydrophilicity, Partition Coefficient, Aggregation and Hydrogen Bonding)

Peptides are amphoteric, so their solubility shifts with pH and is the lowest at their isoelectric point when they carry no net charge. They are typically polar in nature which limits passive diffusion through the lipid membrane. Employing a lipophilic prodrug charge masking approach to change the polarity of hydrophilic peptides, such as with lipid derivatives of cyclic N-methylated hexapeptide containing Arg-Gly-Asp (RGD) showed that the derivatized peptides traversed mucosal membrane via the transcellular pathway in the Caco cell model to enhance polar peptide permeability [77]. Using this modification approach, the permeability of hydrophilic peptides across oral mucosae can be altered. Also, increasing lipophilicity by adding fatty acid chains or other lipophilic moieties has been shown in recent studies to boost buccal absorption, although the correlation with bioavailability remains nonlinear. It is widely recognized that hydrophilic substances primarily cross mucosal barriers through the paracellular pathway, whereas lipophilic drugs tend to be absorbed via the transcellular route [78]. Most peptides solubility varies with pH. It typically decreases near the peptide’s isoelectric point, where it carries no net charge [79]. They are typically highly hydrophilic with low octanol-water partition coefficients, which limit passive diffusion through lipid membranes. However, increasing lipophilicity by adding fatty acid chains or other lipophilic moieties has been shown in recent studies to boost buccal absorption, although the correlation with bioavailability remains nonlinear. Zhang and Bulaj (2012) reviewed the use of lipidation to enhance peptide therapeutics, highlighting fatty acids such as lauric (C12), myristic (C14), palmitic (C16), and stearic acid (C18) as common lipid moieties for conjugation [80]. They discussed lipidated analogs of clinically relevant peptides, including glucagon-like peptide-1 (GLP-1) and insulin, where fatty acid attachment improved metabolic stability, membrane permeability, and extended half-life. The study emphasized that both the fatty acid chain length and the site of lipid attachment critically affect the pharmacokinetic and pharmacodynamic profiles of peptide drugs [80].

The role of hydrogen bonding in peptide absorption has gained renewed attention. Intermolecular hydrogen bonds with water can create a hydration shell that weakens membrane partitioning by increasing peptide polarity and reducing the octanol–water partition coefficient [81]. However, recent studies show that cyclic peptides with strong intramolecular hydrogen bonds, formed during conformational folding, can effectively mask polar groups, lower their exposed polarity and improve passive membrane permeability [82]. Moreover, a study demonstrated that thioamide substitutions (>C=O → >C=S) in macrocyclic peptides reduce desolvation energy and enhance permeability and metabolic stability in vivo by weakening intermolecular hydration [83]. Supporting these results, recent computational and experimental studies demonstrate that peptides engineered to form intramolecular hydrogen bonds and adopt compact structures exhibit significantly enhanced membrane permeability [84]. Merz et al. (2023) developed a high-throughput platform to design and evaluate thioether-cyclized peptides for both target binding and passive membrane permeability [85]. Screening a library of 8448 N-acylated cyclic peptides, they used PAMPA assays to identify candidates with favorable physicochemical profiles. The study demonstrated that select peptides exhibited nanomolar affinity for thrombin and achieved oral bioavailability up to 18% in rats. Key to these findings was the modulation of intramolecular hydrogen bonding and lipophilicity, which improved passive diffusion across epithelial membranes. achieving a key approach to increase effective lipophilicity, reduce hydrogen bonding with water and decrease hydrodynamic radius, thereby enhancing epithelial absorption, solubility and permeability, reinforcing the therapeutic potential of intramolecular hydrogen bonding [85]. Recent study also emphasizes peptide self-association and aggregation, which can impact stability and absorption. For example, insulin’s tendency to aggregate has been shown to be allayed by nonionic-surfactants like Pluronic F-68 and F-127 that form micellar nanoparticles, reducing aggregation and enhancing solubility [86]. Furthermore, buccal formulations such as patches or microneedle-based devices can cause local irritation or toxicity due to prolonged contact with the mucosal tissue [59]. For example, devices designed to physically disrupt the epithelium can lead to discomfort, emphasizing the need for biocompatible, minimally invasive designs. Discomfort and toxicity may result from extended interactions between some delivery methods and the buccal mucosa at the application site. Successful innovative buccal peptide delivery systems must effectively address these limitations.

### 5.4. Electrostatic Charge and Buccal Permeability

The peptide’s overall charge distribution plays a critical role in interaction with mucus networks that impact peptide residence and translocation through the oral mucosa. Molecules, including peptides in their zwitterionic peptides bearing both positive and negative group charges, often exhibit reduced membrane penetration despite favorable partition coefficients, as balanced charges hinder their interaction with epithelial layers. Glutathione 307 Da tripeptide exhibits very low passive membrane permeability. Its charged, hydrophilic character and zwitterionic configuration prevent effective interaction with lipid bilayers and impede diffusion. Fluorescence and NMR-based experiments showed no passive penetration across lipid membranes [87,88]. However, recent advances show that non-classical zwitterions, designed to create internal charge offset and intramolecular hydrogen bonds, can maintain high permeability and low lipophilicity suggesting innovative opportunities to improve peptide transport across the buccal barrier which non-classical zwitterions such as piroxicam an optimized molecular design featuring weak, matched pK_a_ values acting through intramolecular stabilization allows compounds to maintain low lipophilicity in water without sacrificing passive membrane permeability [89]. Also, zwitterionic nanoparticle-coated peptides have shown a 4.5-fold improvement in epithelial uptake using an in vitro cell culture model and effective oral insulin delivery by an in situ rat model, to enhance peptide absorption in the oral cavity [90].

The peptide net charge alone could not fully estimate mucosa transport efficiency. However, in cationic peptides with the addition of hydrophobic residues, the positions of these charged and hydrophobic residues within the peptide govern mucosal transport of the peptide [91]. Modulating the pH of the formulation impacts the degree of peptide ionization and its charge density. At physiological pH, the negatively charged mucosal epithelium preferentially absorbs positively charged peptides. peptides, while negatively charged compounds like insulin are largely excluded from paracellular pathways [92]. Conversely, positively charged peptides such as thyrotropin-releasing hormone demonstrate enhanced uptake via these pathways [93]. Recent approaches include Ion-pairing techniques, which include pairing peptides like insulin with hydrophobic counterions such as bile salts or amino acids that can form neutral complexes that significantly boost mucosal penetration. One study demonstrated a 3–50-fold increase in insulin permeability across buccal tissue when ion-paired with sodium glycodeoxycholate [92].

## 6. Strategies to Enhance Peptide Permeation and Stability via Oral Route

**Figure 3:**
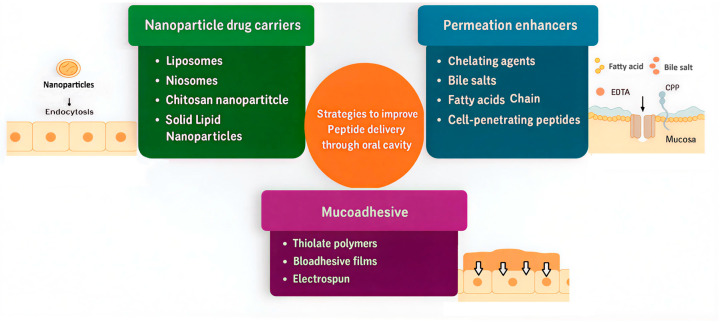
Strategies to enhance peptide delivery into the oral cavity.

Numerous methodologies have been under investigation to surmount challenges associated with the oral absorption of peptides (Figure 3). These cover innovative formulation strategies, chemical coupling with hydrophobic ligands or nanoparticles that exhibit targeted affinities, modifications of amino acid backbone, as well as the utilization of enzyme inhibitors, permeation enhancers, and mucoadhesive polymers [94].

### Formulation Strategies to Enhance Peptide Absorption and Retention in the Mouth Cavity

**A. Permeation enhancers** play a crucial role in improving the bioavailability of peptides administered via the oral mucosa by overcoming the inherent barrier properties of the buccal epithelium. These enhancers primarily operate by transiently disrupting the tight junctions between epithelial cells to increase paracellular transport or by interacting with lipid bilayers to facilitate transcellular drug passage. Among natural permeation enhancers, terpenes such as α-bisabolol that have gained attention due to their ability to interact with mucosal lipids, enhancing drug permeability without affecting tissue integrity, as demonstrated in vitro studies using porcine mucosa in small drug molecules [95]. Chelating agents like EDTA and EGTA act by binding calcium ions that maintain tight junction integrity, thereby transiently increasing intestinal paracellular permeability, their use in oral insulin formulations highlights their clinical relevance [96]. Bile salts, including sodium taurocholate and sodium deoxycholate, function as surfactants reducing epithelial resistance and promoting both transcellular and paracellular permeation [76]. Studies have shown that sodium taurocholate facilitates the transport of large molecules such as fluorescent dextrans (70–150 kDa) across porcine buccal mucosal tissues, which are impermeable, highlighting their potency as enhancers [70]. Medium-chain fatty acids like sodium caprate can fluidize lipid membranes and modulate tight junction proteins such as claudin-5 and enhance peptide uptake while maintaining mucosal safety [97]. Zwitterionic small molecules, including SNAC (sodium N-[8-(2-hydroxybenzoyl) amino] caprylate), improve peptide solubility and permeability, and are key components of marketed oral peptide drugs like Rybelsus^®^, demonstrating their translational potential [98]. Additionally, cell-penetrating peptides (CPPs), such as penetratin analogs like Penetramax, facilitate cellular uptake by inducing endocytosis and modulating tight junction dynamics, as recent research reveals their ability to reversibly open epithelial barriers in a controlled manner [99]. CPPs have been demonstrated to significantly enhance buccal absorption of insulin. Xu et al. (2020) developed a PEGylated low-molecular-weight protamine (LMWP) insulin conjugate designed to improve mucosal permeation and stability. In vitro and ex vivo studies showed increased uptake and transport of insulin across buccal mucosa models, while in vivo experiments in rabbits demonstrated a relative pharmacological bioavailability of 26.9% following buccal administration of the conjugate, markedly higher than unconjugated insulin [100]. The enhanced absorption was attributed to CPP-mediated facilitation of transcellular transport and improved mucoadhesion, which is the reversible adhesion of a dosage form to mucus or epithelial surfaces through hydrogen bonding, electrostatic, hydrophobic, or disulfide interactions in the oral cavity. This prolongs residence and reduces salivary wash-off without inducing mucosal irritation. This work highlights CPP conjugation as a promising strategy for non-invasive insulin delivery via the buccal route. Collectively, these permeation enhancers offer diverse and effective mechanisms to improve buccal peptide delivery, with ongoing research focusing on optimizing their safety and efficacy profiles to advance clinical applications.

**B. Enzyme inhibitors** can overcome the rapid degradation by local proteolytic enzymes, including serine proteases and metalloproteinases (such as aminopeptidase N). To overcome this barrier, enzyme inhibitors have been incorporated into buccal delivery systems to protect peptides from enzymatic cleavage, thereby enhancing their stability and mucosal absorption. For instance, aprotinin is a well-known serine protease inhibitor that has been co-formulated with insulin in thiolated chitosan xerogels, leading to a 70% reduction in enzymatic degradation and a 1.7-fold increase in insulin permeability across human EpiOral™ models and ex vivo sheep buccal mucosa [101]. Another approach involves the use of chitosan–EDTA conjugates, which act by chelating zinc ions essential for metalloprotease activity. Walker et al. (2002) investigated peptidase activity on porcine buccal mucosa and found aminopeptidase N to be the primary enzyme degrading peptides such as Leu-enkephalin, with only 18 ± 9% peptide remaining after 2 h at 37 °C [64]. The study demonstrated that enzyme inhibitors, including amastatin, sodium deoxycholate, and EDTA, significantly reduced peptide degradation. Additionally, sodium deoxycholate acted as a permeability enhancer by disrupting the mucosal barrier, facilitating increased peptide permeation. These findings indicate that combining enzyme inhibitors with permeability enhancers can effectively improve buccal delivery of peptides [64].

Furthermore, thiolated polymers (“thiomers”) have gained prominence for their multifunctional properties: they not only inhibit enzymes by chelating metal cofactors but also enhance mucoadhesion and extend residence time on the mucosa, resulting in improved bioavailability of peptides such as salmon calcitonin and insulin [101]. These findings demonstrate that enzyme inhibitors, particularly when incorporated into multifunctional or polymer-based systems, represent a promising strategy for protecting peptides at the site of buccal absorption and overcoming one of the principal barriers in transmucosal peptide drug delivery.

**C. Mucoadhesive** drug delivery systems have become a central strategy in enhancing the residence time and bioavailability of peptides delivered via the oral mucosa, particularly in the buccal region. These systems are designed to adhere to the mucosal surface, thereby resisting salivary washout and providing a sustained-release environment for therapeutic peptides. Among the most extensively studied mucoadhesive agents are thiolated polymers (thiomers) such as thiolated chitosan, thiolated pectin, and thiolated hyaluronic acid, which can form covalent disulfide bonds with cysteine-rich mucin glycoproteins in the mucus layer [102]. Recent work demonstrated that S-protected thiolated β-cyclodextrin (CD) derivatives increased mucoadhesion several-fold relative to native CD by enhancing both the viscosity and the interpenetration with the intestinal mucosal surface [103]. Similarly, pre-activated thiolated hyaluronic acid showed a 4-fold improvement in mucoadhesion compared to native hyaluronic acid, thus increasing the residence of small drug molecules for potential intraoral application [104]. Another innovative approach involves mussel-inspired bioadhesive films, which utilize catechol chemistry to mimic the strong wet adhesion of marine mussels. These films have demonstrated adhesive forces exceeding 39 kPa and have shown that a sustained release model of small drug molecules entrapped in nanoparticle form is promising to provide controlled peptide release across buccal tissues [105]. Additionally, electrospun mucoadhesive membranes, which present a high surface area and interwoven fibrous architecture, facilitate strong physical interlocking with the mucus and were recently shown to maintain insulin activity and release over an 8 h window [106]. For example, Amer et al. (2025) developed niosomal formulations of vancomycin hydrochloride, a glycopeptide antibiotic, incorporated into polyvinyl alcohol (PVA) fast-disintegrating oral films [107]. These films demonstrated small particle sizes, high drug encapsulation efficiency, rapid disintegration (~105 s), and effective antibacterial activity against *Bacillus subtilis*. This study highlights how combining nanocarriers with mucoadhesive polymers like PVA can enhance the oral delivery potential of macromolecular drugs that typically suffer from poor permeability and stability issues [108]. These hybrid films not only adhere effectively to the buccal mucosa but also resist enzymatic degradation and offer controlled peptide release. Overall, mucoadhesive strategies, including both natural and synthetic polymers like PVA, are integral in the advancement of oral mucosal peptide delivery platforms, combining prolonged contact time, improved stability, and increased therapeutic efficacy.

## 7. Nanoparticulate Strategies for Buccal and Sublingual Peptide Delivery

### 7.1. Rationale for Nanoparticle Use in Oromucosal Peptide Delivery

**Figure 4:**
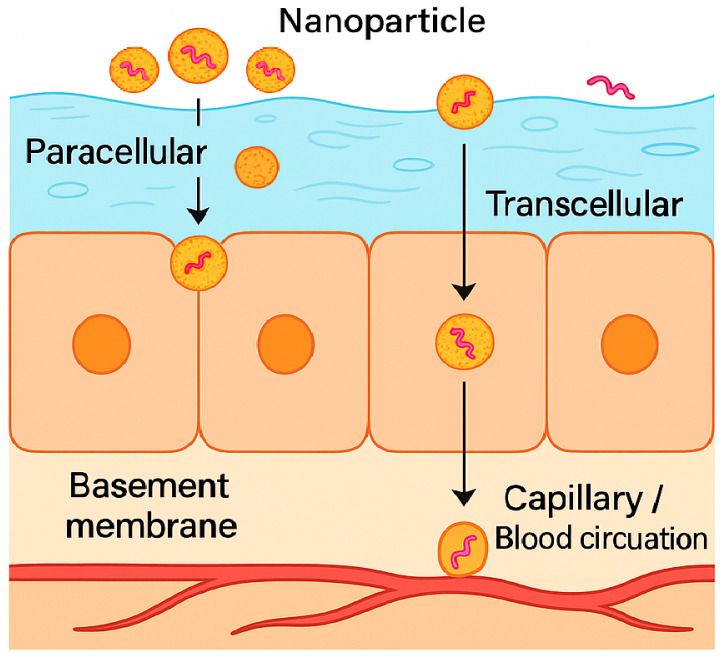
Epithelial cell penetration through nanocarrier systems.

Peptide therapeutics face many obstacles in the oral cavity. Continuous saliva flow dilutes and removes the applied dose. The mucus layer, which is rich in mucins, slows diffusion by trapping large molecules. Tight junctions between epithelial cells restrict movement through the spaces between cells. Enzymes in saliva, such as aminopeptidases and endopeptidases, degrade peptides before they reach the epithelial surface. Together, these factors shorten residence time and lead to low and variable absorption across the buccal and sublingual mucosa, even for small peptides with suitable properties [38]. One strategy to enhance peptide delivery through the oral cavity is the incorporation of peptide molecules into nanoparticle carriers, which can protect them from enzymatic degradation and facilitate their transport across the oral epithelial barriers (Figure 4) [109,110,111]. Nanocarriers have drawn a lot of interest among the many techniques that are accessible [112].

Nanoparticles, typically submicron carriers with diameters of 300 nm or less, are promising systems for the oral delivery of peptides such as insulin due to their high stability and protective properties. Polymeric nanoparticles, in particular, can encapsulate both hydrophilic and hydrophobic molecules and release them from the polymeric matrix in a controlled manner. This sustained and regulated release not only shields peptides from enzymatic degradation but also prolongs their residence time and enhances their effective concentration at the absorption site. Consequently, nanoparticle systems can significantly improve the bioavailability of therapeutic peptides that are delivered via the oral mucosa [113,114]. Beyond basic protection and controlled release, nanoparticle carriers provide several adjustable functions that conventional solutions, gels, or tablets do not offer. Vesicular and polymeric systems shield the peptide from salivary enzymes and dilution until the carrier reaches the epithelial surface. Surface modification using chitosan or thiomer coatings, polyethylene glycol or zwitterionic layers, and other layered shells can be tuned to strengthen mucoadhesion and extend residence time, or to promote movement through mucus when deeper access is needed. By adjusting composition and size, the carrier can also be directed toward transcellular uptake through endocytosis and subsequent transcytosis, a pathway favored by lipid based particles. In selected cases, and only when safety and reversibility are demonstrated, permeation enhancers may be included to produce a brief increase in paracellular transport across tight junctions [48].

Evidence from oromucosal models supports these principles. Neutral nanoparticles with an intermediate size of about 200 nm penetrate more deeply and remain longer within the buccal epithelium, for example, within the microplicae, than smaller particles of 25 to 50 nm. This pattern aligns with stronger interactions observed in human oral epithelial cell models TR146. When these carriers are incorporated into mucoadhesive dosage forms, including films, patches, or sprays that form thin films in the mouth, a dose that would otherwise be rapidly cleared by enzymes becomes retained, protected, and able to interact effectively with the epithelium [115]. Taken together, the evidence shows that nanoparticles are not just small containers but adaptable tools for oromucosal delivery. They position protective microenvironments at the epithelial surface and pair this protection with adhesion or mucus penetration. They help maintain effective concentration at the mucosa despite continuous saliva flow. They also provide adjustable pathways for transport across tight epithelial barriers. These features offer a clear basis to prioritize nanoparticle systems over conventional liquid or semi-solid dosage forms for peptide delivery in the oral cavity [48].

### 7.2. Nanoparticle Classes and Their Comparative Potential

#### 7.2.1. Lipid-Based Nanoparticles

Lipid systems are the most established platforms for oromucosal delivery due to their combining protection from enzymes with affinity for cell membranes and adjustable surface chemistry. There are different type of Lipid-based vesicles, such as liposomes, transferosomes and niosomes. Conventional liposomes can encapsulate peptides, and their surfaces can be modified with polyethylene glycol, zwitterionic groups, or mucoadhesive coatings. The bilayer is flexible under shear, which improves contact at the mucus epithelium interface and supports endocytic uptake when residence time is controlled by a film or a patch. Solid lipid nanoparticles and nanostructured lipid carriers add matrix stability and protect against salivary proteases. In practice, peptide loading can be increased by ion pairing or by remote loading and embedding these carriers in mucoadhesive dosage forms helps resist washing off. Route focused studies support these design choices and stress that stability after processing, for example, after drying into films or sprays, should be verified to preserve particle size and surface properties [48]. As an illustration, chitosan-coated liposomes carrying salmon calcitonin (sCT) peptide have been reported to increase mucoadhesion and epithelial uptake, with in vivo (rat) studies showing enhanced bioavailability and prolonged plasma exposure in addition to a consistent residence-time and protection with the mechanisms outlined above [48].

#### 7.2.2. Niosomes

Niosomes nanoparticles are vesicular made from nonionic surfactants and cholesterol. They offer practical stability advantages over phospholipid liposomes because they are less prone to oxidative and hydrolytic degradation. At the same time, they allow flexible surface modification, including polyethylene glycol, chitosan coatings, and ligand or cell penetrating peptide decoration. In buccal and sublingual delivery, niosomes perform best when they are incorporated into dosage forms such as films, gels, or sprays that form thin films in the mouth, since simple suspensions are easily washed away by saliva and may be swallowed. The type of surfactant and the amount of edge activator strongly affect bilayer rigidity, leakage, and tissue tolerance, so fair comparisons require matched surfactant levels and controlled edge activator ratios. Reports of higher permeability should always be evaluated alongside safety indicators, in particular, recovery of transepithelial electrical resistance and relocalization of tight junction proteins, to confirm that effects are reversible on oral epithelium [116]. Recent route focused reviews describe these formulation factors and their impact on buccal performance [109]. In line with these principles, PEG-coated niosomes encapsulating thymopentin (TP5) have shown high encapsulation efficiency and endocytosis-mediated cellular uptake with enhanced in vitro activity, and vancomycin-loaded niosomes have exhibited improved stability and cellular delivery suitable for mucosal peptide therapy [107,117].

#### 7.2.3. Polymeric Nanoparticles

Polymeric carriers, including chitosan and thiolated chitosan, PLGA, and layer-by- layer polyelectrolyte capsules, offer advantages that complement lipid systems. Poly lactic co-glycolic acid (PLGA) is a biodegradable copolymer widely used in medical products. PLGA protects sensitive peptide cargo and supports controlled release, and its surface can be coated with chitosan or polyethylene glycol to adjust how the particles interact with the oral mucosa. Chitosan and thiomers provide strong mucoadhesion through electrostatic and hydrogen bonding, and they can produce a brief and reversible opening of tight junctions through ionic effects and exchange with cysteine rich mucins. This can increase paracellular transport of peptides. For translation, surface charge should be balanced. A mildly positive zeta potential, about +5 to +20 millivolts after hydration, usually improves adhesion and uptake without causing irritation. When higher cationic density is needed, mixed or patchy coatings, such as chitosan combined with polyethylene glycol or zwitterionic groups, can reduce cytotoxicity. Evidence from recent studies on thiolated chitosans supports these choices, and models, including TR146 cells and organotypic oral mucosa, that are suitable to confirm both increased permeability and recovery of barrier function. Recovery is shown by restoration of transepithelial electrical resistance (TEER) to the starting value after washout, and by relocalization of the tight junction proteins claudin and occludin, meaning that these proteins return to the cell borders in microscopy images. Together, TEER restoration and relocalization of claudin and occludin show that any increase in permeability is temporary and safe [118,119]. In applied contexts, chitosan–TPP insulin nanoparticles have produced hypoglycemic effects in diabetic rats, attributed to strong mucosal retention and improved epithelial translocation [120,121], and thiolated chitosan–insulin systems have increased transepithelial transport across Caco-2 monolayers, indicating the potential for paracellular facilitation where justified [122]. Together, these elements improve peptide stability and transport during the intended residence time.

#### 7.2.4. Hybrid Responsive Nanocarriers

Lipid polymer hybrids combine a lipid core that interacts well with cell membranes with a polymer shell that provides mucoadhesion and stability Likewise, potent therapeutic compound with high aqueous solubility, was successfully encapsulated into polymer–lipid hybrid nanoparticles (PLHNs). The system consisted of a polymeric core formed by PLGA–PEG–PLGA triblock copolymers and a surrounding lipid shell composed of lecithin and cholesterol. Incorporation of drug into these polymer–lipid hybrid nanoparticles resulted in markedly improved entrapment efficiency compared with conventional polymeric nanoparticles [123]. In buccal and sublingual delivery this design often outperforms single material carriers of the same size and charge because it links residence at the surface with efficient cell interaction. Triggers that work under salivary conditions are the most practical, including hydration driven swelling of films for sustained release at the surface, enzyme sensitive linkers that uncover the peptide or a permeability motif, and thiol disulfide exchange in thiolated coatings. Large changes in acidity are less useful because saliva is strongly buffered. Composite dosage forms such as electrospun mats can carry high loads and break down in a controlled way, but they raise manufacturing demands and require reliable reproducibility of particle properties after processing [123]. Studies that reflect the intended route, including deformable vesicles for insulin in animals and nanoparticle systems embedded in gels or films, show that residence time and interaction with the epithelium, rather than diffusion alone, determine the delivered dose in the oral cavity [124].

## 8. Mechanisms and Determinants of Oromucosal Nanoparticle Performance

### 8.1. Local Microenvironments for Saliva and Mucus

After nanoparticle contact with saliva, the behavior is driven by electrolyte strength, mucins, and the rapid formation of a salivary pellicle (thin protein film derived from saliva). Ions reduce the electrical double layer, mucins can bridge particles, and absorbed peptide form a dynamic surface layer of biomolecules known as protein corona that could change adhesion and cell interaction of the peptide [125]. Polyethylene glycol or zwitterionic coatings limit hydrophobic and electrostatic sticking and enable motion through mucus; brush-like densities are needed to avoid immobilization, while subcritical coverage can increase mucoadhesion. Comprehensive reporting of physicochemical parameters such as grafting density, polymer conformation, and particle mobility obtained from multiple-particle tracking is essential to support translational claims for nanocarrier systems. For instance, surface grafting density of polymers such as polyethylene glycol (PEG), polyvinylpyrrolidone (PVP), or chitosan markedly influences corona formation, steric stabilization, and mucosal adhesion. Detailed characterization of polymer chain conformation whether brush-like, mushroom, or collapsed provides insight into the accessibility of surface ligands and interaction with biological barriers. Coupling these measurements with multiple particles tracking dyes in relevant biological media (e.g., mucus or epithelial models) enables correlation between nanoscale mobility, interfacial behaviors and in vivo translational potential [126]. Because saliva clears formulations quickly, any extended surface residence would increase the peptide permeability. Adhesion-focused designs will resist wash-off and the nanoparticle slip-through and deliver carriers to the cell surface [109,127]. Saliva contains diverse proteases whose activity varies between individuals and overtime. Encapsulation of the peptide alone is often not sufficient. Local microenvironments that adjust water activity or pH, and barrier layers that slow enzyme access, can extend peptide half-life at the surface. If protease inhibitors are used, their route suitability and any effect on pellicle formation should be shown. Peptide integrity should be confirmed in whole saliva and, when possible, by relevant activity assays [115].

### 8.2. Nanoparticle Size, Distribution, and Surface Charge

Evidence from route-relevant models points to an intermediate nanoparticle size window as most effective for buccal delivery. Yang et al. showed that deformable nanovesicles loaded with insulin and sized around 220 nm produced the highest buccal absorption and a marked glycemic reduction in rabbits, whereas larger vesicles near 400 nm displayed lower permeability and bioavailability. Taken together with related datasets, these findings indicate that nanoparticles in the 200–300 nm range provide a practical balance between epithelial penetration, avoidance of mucus immobilization, and systemic delivery efficiency for buccal applications [128]. Beyond the mean diameter, polydispersity and particle shape also influence how carriers lodge within epithelial microplicae under shear; accordingly, studies should report number-weighted sizes and re-measure particles after drying and rehydration, since processing can alter both size and interfacial properties [115]. Nanoparticles with a positive (cationic) surface charge can enhance mucoadhesion by interacting electrostatically with negatively charged mucin and epithelial membranes, potentially facilitating paracellular transport. However, excessive or uniform cationic charge may cause membrane irritation or cytotoxicity. In contrast, nanoparticles with localized or patchy positive charges on a well-hydrated surface for example, chitosan-coated PEGylated nanoparticles can maintain sufficient adhesion and permeability while reducing irritation compared with uniformly high-zeta-potential systems. When a paracellular route is intentionally targeted, a brief and reversible opening of tight junctions is required in which calcium chelators can lower extracellular calcium and loosen cadherin complexes, and medium-chain fatty acids or bile salts can disturb membrane order and temporarily reorganize claudin and occludin. Any rise in permeability should occur together with a drop in transepithelial electrical resistance (TEER) and be followed by full recovery of both. Strong evidence, therefore, couples increased apparent permeability with TEER recovery, imaging that shows tight-junction proteins first disperse and then return to cell borders, and a washout step that restores baseline [119,129].

### 8.3. Nanoparticle Cellular Uptake and Trafficking

Lipid-based nanoparticles such as liposomes, solid lipid nanoparticles, nanostructured lipid carriers, and elastic vesicles can be taken up by cells through several mechanisms, including clathrin mediated endocytosis, caveolae mediated endocytosis, or macropinocytosis. Macropinocytosis is a non-selective, actin driven process where the cell membrane folds outward to engulf surrounding fluid and particles into large vesicles inside the cell. Unlike receptor mediated endocytosis, which requires specific ligand binding, macropinocytosis allows bulk uptake of nanoparticles without the need for targeting molecules. This process helps larger or more flexible nanoparticles enter cells efficiently, improving intracellular trafficking and drug delivery. In contrast, earlier transport mechanisms such as paracellular diffusion or mucus penetration occur mainly outside the cells. Macropinocytosis therefore offers a more active, cell mediated route that enhances the entry and bioavailability of nanoparticle encapsulated drugs. Matrix lipids, the amount and type of edge activator, and the surface coating together set both the extent and the route of uptake and the balance between transport and cellular stress. In human TR146 cells, composition-dependent intracellular trafficking supports designing carriers for productive transcytosis while limiting adverse signals.

Penetratin-conjugated liposomes increased salmon calcitonin permeation across TR146 layers and ex vivo porcine buccal tissue, demonstrating peptide-assisted transcellular transport under route-relevant conditions. Ligand-directed strategies (e.g., folic acid, lectins) and membrane-mimicking lipid domains can promote receptor-mediated or lipid-raft internalization, and in some systems membrane fusion. Mechanistic proof should combine pathway inhibition, co-localization with Rab5, Rab7, and lysosome markers, and detection of intact peptide on the basolateral side by liquid chromatography–mass spectrometry, with a functional bioactivity readout when endosomal escape is claimed [130,131].

### 8.4. Device Perspective

Nanoparticles are rarely delivered as simple suspensions, as they tend to disperse quickly and are washed away by saliva before significant absorption occurs. More effective delivery can be achieved through mucoadhesive films, patches, or sprays that form thin films over the mucosa. These dosage forms maintain intimate and prolonged contact with the epithelium, sustain a local concentration gradient, and minimize drug loss due to dilution or swallowing [48]. Film-forming sprays distribute carriers evenly and create uniform in situ films that improve stability and onset [132]. As a route-relevant example, vancomycin-loaded niosomes in mucoadhesive films achieve local antimicrobial action in the oral cavity and provide a basis for possible systemic uptake [107]. Processing steps such as lyophilization, casting, and spraying can alter lipid crystallinity, brush hydration, and peptide location; characterization must therefore be performed on the final dosage form in saliva-mimicking media, with batch reproducibility documented [48,130].

### 8.5. Comparative Design and Human Evidence

Across buccal and sublingual models, three design elements persist: integration into a device to secure residence at the mucosal surface, an intermediate particle size that matches the epithelial microarchitecture, and transcellular engagement that balances uptake with tolerability. These features explain why surface-engineered vesicles or hybrid carriers of approximately 200 nm, maintained in position by a dosage form, repeatedly perform well in oromucosal designs. Representative examples include penetrating-conjugated liposomes for salmon calcitonin and deformable vesicles for insulin, reinforcing the principle that composition, together with residence time controls performance [109,133]. For systemic use, early human studies with buccal insulin sprays, though not nanoparticulate, showed measurable effects and rapid onset but variable outcomes; reviews conclude that exposure is achievable yet inconsistent, with relative bioavailability usually low to moderate and highly dependent on formulation and use conditions. Human data for nanoparticle-enabled insulin remain limited compared with animal studies, but feasibility shown by sprays and strong preclinical work on deformable nanovesicles and nanoparticle-in-film systems support continued development of higher-payload, longer-contact nano-in-device strategies [134]. Also, Insulin has been formulated as glycan-coated gold nanoparticles embedded in a thin mucoadhesive polymer film for transbuccal delivery, known as Midaform™ Insulin PharmFilm^®^. The glycan corona stabilizes insulin in saliva, the gold core provides a reproducible nanoscale scaffold, and the film maintains residence under salivary flow while releasing the nanoparticle insulin complex at the mucosal surface. First-in-human studies showed rapid pharmacodynamic responses with acceptable local tolerability and the program advanced from Phase 1 to Phase 2a initiation [135]. Reported exposure was acceptable and variable, which is consistent with current oromucosal experience, but the case remains a rare clinical example of a nanoparticulate peptide delivered via the buccal mucosa.

### 8.6. Safety and Manufacturability Standards

Efficacy must be judged together with safety. Any reported increase in permeability with enhancers or cationic surfaces should be paired with proof of reversibility (transepithelial electrical resistance recovery after washout and return of ZO-1, claudin, and occludin to cell borders on microscopy). TR146 cell culture models are useful for the mechanism but should be complemented by ex vivo porcine buccal tissue under flow or tilt to improve validity. Composition can raise uptake without raising cellular stress, supporting composition-guided optimization rather than simply increasing positive charge [136]. In parallel, inorganic and carbon-based nanoparticles require caution in the oral cavity due to potential cytotoxicity and limited biodegradability. Examples silica nanoparticles, and carbon nanotubes, which may accumulate in tissues or induce inflammatory responses. These safety concerns can limit their clinical translation compared with more biocompatible organic carriers [137]. Most oromucosal products will not deliver nanoparticles as simple suspensions. Drying, casting, and spraying can change particle size, zeta potential, crystallinity in solid lipid carriers, and peptide location; characterization after processing in saliva-mimicking media is therefore a core performance requirement. Strong chemistry, manufacturing, and controls practices encompass design, production, and quality assurance measures that ensure translational reliability. For example, structural or surface modifications such as PEGylation, chitosan coating, or lipid shell incorporation can improve nanoparticle stability and mucoadhesion. Manufacturing practices like controlled solvent evaporation, microfluidic mixing, or high-pressure homogenization ensure uniform particle size and drug loading. Controls include verifying batch-to-batch reproducibility, monitoring particle integrity and zeta potential, and testing stability in biologically relevant media such as simulated saliva. Together, these measures distinguish robust, clinically credible nanoparticle systems from fragile laboratory prototypes [48].

### 8.7. Limitations

Despite encouraging findings, several factors limit the translation of nanoparticle delivery across the oral mucosa. First, the evidence base is small and heterogeneous. Studies differ in tissues, devices, and readouts, which hinder comparison and can inflate effects when residence time is not controlled or reported [48]. Second, common in vitro surrogates such as TR146 cell model are useful for studying mechanism but do not reproduce the stratified buccal epithelium; confirmation by using ex vivo porcine buccal tissue under flow or tilt is recommended. Third, the oral microenvironment varies in rheology, ionic strength, and protease activity, which alters nanoparticle stability, protein corona formation, and peptide half-life; claims based only on buffers or a single artificial saliva are weak [126]. Fourth, strategies that open tight junctions or use strongly cationic surfaces must demonstrate reversibility and tolerability, including recovery of transepithelial electrical resistance and relocalization of ZO-1, claudin, and occludin, since effective levels of chelators or medium chain fatty acids can compromise barrier integrity; intestinal safety does not guarantee oral safety [138]. Fifth, manufacturability is a practical bottleneck. Most clinical candidates will be films, patches, or sprays that form films, and processing can change size, zeta potential, crystallinity in solid lipid carriers, and peptide location; route credible chemistry, manufacturing, and controls therefore require recharacterization after processing and in defined saliva [139]. Early human studies with buccal insulin showed measurable but variable exposure with generally low to moderate relative bioavailability, indicating the need for higher load devices, longer controlled contact, and dose-sparing peptide design [140]. Overall, progress is most likely when studies adopt standardized reporting of delivery site, surface area, residence time, and swallowing control. Formulation performance should be evaluated using saliva-aware stability and residence assays, such as in vitro dissolution in simulated saliva, ex vivo mucosal retention, or fluorescent particle tracking in mucosal tissue. Reversibility packages can be assessed in oral models by monitoring epithelial barrier recovery, for example, measuring transepithelial electrical resistance (TEER) or tight junction protein relocalization after nanoparticle exposure. Finally, the processed state of the final device should be characterized using parameters such as particle size and distribution, zeta potential, film thickness, drug content uniformity, and mechanical strength. Together, these measures ensure that oral nanocarrier devices are robust, reproducible, and translationally credible [48].

## 9. Promising Advances in Nanocarrier-Based Peptide Delivery via the Oral Cavity and Mucosa

The delivery of peptides via the buccal or sublingual route benefits substantially from the integration of nanocarriers within thoughtfully designed dosage forms such as mucoadhesive gels, films, tablets, sprays or pastes. This dual strategy not only influences the protective and permeation-enhancing capabilities of nanoparticles for example (liposomes, niosomes, solid lipid nanoparticles, polymeric nanoparticles), but also capitalizes on the controlled release, improved mucosal adhesion and increase the residence time provided by the matrix [48]. Recent reviews report that nanoparticle-in-matrix systems such as lipid or polymeric NPs embedded in gels, films, or solid tablets consistently demonstrate higher bioavailability and prolonged release profiles compared to formulations lacking nanocarriers [141]. In addition, mucoadhesive buccal films and patches loaded with lipid NPs achieve intimate mucosal contact, significantly extend retention time and protect embedded peptides from enzymatic degradation yielding better uptake compared to traditional formulations Table 4. Meanwhile, film-forming sprays allow even distribution of nanocarriers across the localized area, forming a uniform in situ film that enhances stability and rapid onset. Incorporating nanocarrier systems such as liposomes, niosomes, or polymeric nanoparticles into designed dosage forms like mucoadhesive films, gels, sprays, or tablets significantly improves peptide delivery via the oral mucosa.

## 10. Conclusions

The effective delivery of therapeutic peptides via the oral cavity by sublingual or buccal route presents both opportunities and challenges over peptide delivery to the intestines. Intrinsic properties of most peptides which are polar with relatively large size and labile in nature are still an issue when administering through this route due to the presence of several barriers. They include enzymatic degradation by proteases or peptidases, low permeability across the mucosal epithelium, and rapid clearance due to salivary dynamics. In addition, drug absorption is affected by its location within the oral cavity has added complexity that necessitate design of delivery systems that take these factors into consideration. Structural or conformation modification, co-administration of enzyme inhibitors, addition of permeation enhancers or mucoadhesive polymers and nanotechnology offers a powerful toolkit to overcome these limitations for buccal and sublingual peptide delivery in non-invasive manner. Apart from providing protection, nanocarriers and nanoplatforms that have been engineered to appropriate size and surface properties have demonstrated an enhancement in buccal peptide delivery using in vitro or ex vivo models. Despite many successful examples, the clinical translation of peptide nanocarriers for administration via oral cavity remains sparse, this gap reflects the need for more research to be performed to establish the efficacy and safe usage of nanocarrier systems.

## Figures and Tables

**Table 1:** Selected Peptide Therapeutics with Clinical Use: Routes and Molecular Weights.

Peptide	MW (Da)	Therapeutic Uses	Dosage Form
Insulin	~5800	Diabetes mellitus	Injection (subcutaneous)
GLP-1 analogs (Liraglutide, Semaglutide)	~3751 (Liraglutide)	Type 2 diabetes, obesity	Injection (subcutaneous and oral)
Oxytocin	1007	Labor induction, postpartum bleeding	Injection, IV infusion
Vasopressin	1084	Diabetes insipidus, bleeding control	Injection, nasal spray
Calcitonin	3432	Osteoporosis, hypercalcemia	Injection, nasal spray
Enfuvirtide	4492	HIV treatment (fusion inhibitor)	Injection (subcutaneous)
Leuprolide	1209	Prostate/breast cancer, endometriosis	Injection (IM, SC), implant
Desmopressin	1069	Diabetes insipidus, bleeding disorders	Nasal spray, oral
Somatostatin/Octreotide	1637 (Somatostatin)/1019 (Octreotide)	Acromegaly, neuroendocrine tumors	Injection (IM, SC), depot injection
Exenatide	4186	Type 2 diabetes	Injection (SC), extended-release injection
Bremelanotide	1024	Hypoactive sexual desire disorder	Injection (SC)
Thymosin Alpha-1	3108	Immunomodulation (Hepatitis B, C)	Injection (SC, IM)
BPC-157	1419	Experimental: wound healing, inflammation	Experimental; injection (SC or IM)
Glucagon	3485	Severe hypoglycemia emergency treatment	Injection (IM, SC, IV)
Melanotan II	1025	Investigation: skin tanning, sexual dysfunction	Injection (SC)
Thyrotropin-releasing hormone (TRH)	362	Diagnostic for pituitary disorders; neuroregulation	Injection (IV)
Bivalirudin	2180	Anticoagulant	Injection (IV)
Goserelin	1282	Prostate cancer, breast cancer, endometriosis	Implant, injection (SC)
Follitropin alfa	~30,000	Fertility treatment (FSH analog)	Injection (SC, IM)
Vasopressin analog (Terlipressin)	1056	Acute variceal bleeding, septic shock	Injection (IV)
Daptomycin (cyclic lipopeptide)	1620	Gram-positive bacterial infections (MRSA, VRE)	Injection (IV)
Colistin (Polymyxin E)	~1155	Multidrug-resistant Gram-negative infections	Injection (IV, IM)
Teicoplanin	~1883	Gram-positive infections, including MRSA	Injection (IV, IM)
Gramicidin	~1900	Topical infections	Topical (ointment)
Vancomycin (glycopeptide)	1449	Serious Gram-positive infections	Injection (IV), Oral (for C. diff)
Pexiganan (synthetic magainin analog)	2249	Topical diabetic foot infections (under evaluation)	Topical cream
Bacitracin	~1422	Topical antibacterial for minor skin infections	Topical ointment
Nisin	~3350	Food preservation, investigational clinical use	Topical, experimental

**Table 2:** Comparison of the intestinal tract and oral cavity.

Features	Intestinal Tract	Oral Cavity
**Epithelium cell type**	Single-layer columnar epithelium with mucus layer (non-keratinized)	Stratified squamous epithelium (keratinized or non-keratinized)
**Epithelial Thickness**	Thin (20–30 µm) [55]	Thicker (100–200 μm) for non-keratinized epithelium
**Surface Area**	Very large (due to villi and microvilli)	Limited surface area
**Mucus Layer**	Variable thickness and the thinnest in small intestine	Thinner mucus layer [56]
**Enzymatic Barriers**	High activity of digestive enzymes (proteases, peptidases) in lumen and brush border	Proteolytic enzymes are present but less extensive than GIT lumen
**pH Environment**	Variable, acidic (stomach pH 1–3), neutral to basic in intestines	Neutral pH (6.5–7)
**First-Pass Metabolism**	Significant hepatic first-pass metabolism	Bypasses hepatic first-pass metabolism
**Transit Time**	Rapid and variable; exposure limited by gastric emptying and intestinal transit	Short residence time and increase duration of stay with special aids
**Absorption Route**	Transcellular, paracellular and endocytic	Transcellular, paracellular and some endocytic [57]
**Salivary Washout**	Not applicable	Present, reduces drug residence time
**Bioavailability**	Low	Low
**Accessibility and Control**	Anatomically deep within the body, limiting ease of access and direct control over administration conditions	Easily accessible and visible, allowing for precise administration and better control of local environment
**Advantages**	Large surface area and non- invasive	Potential for rapid onset of drug action and may be removed, less metabolism
**Disadvantages**	Harsh enzymatic and acidic environment, first-pass metabolism	Small surface area and presence of salivary clearance

**Table 3:** Comparative permeability of macromolecules across oral mucosa models. Note: Papp is apparent permeability.

Route/Model	Compound and MW	Permeability Enhancer	Permeability/Outcome	Reference
**Buccal (in vivo, pig)**	FD-4 (4 kDa)	without enhancement	Bioavailability 1.8%	Hoogstraate et al., 1994
**Buccal (in vivo, pig)**	FD-4 (4 kDa)	10 mM sodium glycodeoxycholate (GDC)	Bioavailability increased to 12.7%	[69]
**Sublingual (porcine)**	FD70 dextran (~70 kDa)	without enhancement	Papp 2 × 10^−10^ cm/s extremely low permeability	Berka et al., 2019
**Sublingual (porcine)**	FITC-BSA (66 kDa) As nanofiber mats	without enhancement	Papp1 × 10^−7^ cm/s 1000× faster than FD70 dextran (~70 kDa)	[72]
**Esophageal porcine mucosa (a buccal model)**	FITC-dextrans (4–70 kDa)	without enhancement	P_app ≤ 10^−7^ cm/s for >40 kDa FD-70 and FD-150 did not cross the membrane in detectable amounts	Fantini et al., 2022 [70]
**Esophageal porcine mucosa (a buccal model)**	FITC-dextrans + enhancer	+caprylic acid/taurocholate	P_app significantly increased, enabling >10 kDa transport	
**Buccal (TR146 cells)**	Salmon calcitonin (3.4 kDa)	+12.2 µM penetratin (CPP)	5.5× increase in permeability over passive	Keum et al., 2020
**Buccal (porcine tissue)**	Salmon calcitonin (3.4 kDa)	+12.2 µM penetratin (CPP)	93.7× increase in permeability over passive	[71]

**Table 4:** Nanocarrier Systems intended for Buccal and Sublingual Peptide Delivery.

Nanosystem	Peptide	Key Benefits	References
**Liposomes**	Salmon calcitonin (sCT)	Cell-penetrating peptide liposomes enhance epithelial uptake and mucoadhesion	[131]
**Niosomes**	Thymopentin (TP5), Vancomycin, Nisin	Pegylated niosomes improve uptake via endocytosis; high encapsulation	[107,117]
**Chitosan-based PEG-b-PLA nanoparticles**	Insulin	Strong mucoadhesion, protection from enzymatic degradation	[120]
**Thiolated chitosan NPs**	Insulin, GLP-1	Open tight junctions, enhanced paracellular transport	[122]

## Data Availability

The original contributions presented in this study are included in the article. Further inquiries can be directed to the corresponding author.
